# Influence of Polymer Shell Molecular Weight on Functionalized Iron Oxide Nanoparticles Morphology and In Vivo Biodistribution

**DOI:** 10.3390/pharmaceutics14091877

**Published:** 2022-09-05

**Authors:** Roxana Cristina Popescu, Bogdan Ştefan Vasile, Diana Iulia Savu, George Dan Mogoşanu, Ludovic Everard Bejenaru, Ecaterina Andronescu, Alexandru Mihai Grumezescu, Laurenţiu Mogoantă

**Affiliations:** 1 Department of Life and Environmental Physics, Horia Hulubei National Institute for Physics and Nuclear Engineering, 30 Reactorului Street, 077125 Măgurele, Romania; 2 Department of Science and Oxide Materials and Nanomaterials, University Politehnica of Bucharest, 1–7 Gheorghe Polizu Street, 011061 Bucharest, Romania; 3 Department of Bioengineering and Biotechnology, Faculty of Medical Engineering, University Politehnica of Bucharest, 1–7 Gheorghe Polizu Street, 011061 Bucharest, Romania; 4 Department of Pharmacognosy & Phytotherapy, Faculty of Pharmacy, University of Medicine and Pharmacy of Craiova, 2 Petru Rareş Street, 200349 Craiova, Romania; 5 Academy of Romanian Scientists, 3 Ilfov Street, 050044 Bucharest, Romania; 6 Research Institute of the University of Bucharest—ICUB, University of Bucharest, 050657 Bucharest, Romania; 7 Research Center for Microscopic Morphology and Immunology, University of Medicine and Pharmacy of Craiova, 2 Petru Rareş Street, 200349 Craiova, Romania

**Keywords:** magnetite nanoparticles, polyethylene glycol, morphology, in vivo, biodistribution

## Abstract

Iron oxide nanoparticles (IONPs) have been extensively used in different biomedical applications due to their biocompatibility and magnetic properties. However, different functionalization approaches have been developed to improve their time-life in the systemic circulation. Here, we have synthesized IONPs using a modified Massart method and functionalized them in situ with polyethylene glycol with different molecular weights (20 K and 35 K). The resulting nanoparticles were characterized in terms of morphology, structure, and composition using transmission electron microscopy (TEM) and selected area electron diffraction (SAED). In vivo biodistribution was evaluated in Balb/c mice, the presence of IONP being evidenced through histopathological investigations. IONP morphological characterization showed a change in shape (from spherical to rhombic) and size with molecular weight, while structural characterization proved the obtaining of highly crystalline samples of spinel structured cubic face-centered magnetite. In vivo biodistribution in a mice model proved the biocompatibility of all of the IONP samples. All NPs were cleared through the liver, spleen, and lungs, while bare IONPs were also evidenced in kidneys.

## 1. Introduction

Iron oxide nanoparticles (IONPs), namely magnetite, have attracted much interest during the last 20 years, especially in the area of medical applications such as diagnosis [1,2,3,4], cancer treatment [5,6,7,8,9], iron deficiency supplements [10,11,12,13], and antimicrobial [14,15] or tissue engineering [16,17,18] where clinically available options have been developed. For these purposes, different functionalization has been applied to IONPs to improve their biocompatibility and reduce their aggregation [19,20]. First, due to their hydrophobic surface and high surface reactivity, bare magnetite NPs are prone to interaction with serum proteins and cells of the reticuloendothelial system, leading to the formation of blood clogs or rapid elimination from the blood system [21]. In the second place, the high surface area and magnetic properties can lead to rapid agglomeration of the bare magnetite NPs in the absence of a protecting coating.

In this study, we have prepared IONPs and functionalized them with polyethylene glycol (PEG) with two different molecular weights (20 K and 35 K, respectively). This was carried out using a simple modified Massart method which was previously proposed by our group [22]. The method allows for obtaining a high yield of in situ conjugated Fe_3_O_4_ nanosystems directly with active substances. These nanoparticles have shown good results in applications such as anticancer drug delivery [22,23,24], radiosensitization [23,24], and in microbial combating [25,26] in vitro. The novelty of this study comes from using the synthesis method to add a polymer layer for the purpose of biocompatibility improvement. The research aims to evaluate the influence of the polymer molecular weight on the morphology, structure, in vivo biocompatibility and biodistibution of the resulting nanoparticles. For morphological investigations, both transmission electron microscopy (TEM) and high-resolution TEM (HR-TEM) were employed, while the structural investigations were carried out using selected area electron diffraction (SAED) and HR-TEM. The biocompatibility of the IONPs was evaluated in vivo using a biodistribution study carried out for a mice model.

Iron oxide nanoparticles have been extensively investigated over the past few years in terms of in vivo behavior [27,28,29,30] However, the mechanisms of interaction between the NPs and the biological systems has still not been made clear [27]. Here, the originality of this study is mainly given by the time frame that is covered by the biodistribution investigation, which exceeds the time point of clinical application (less than 24 h).

## 2. Materials and Methods

For the synthesis of the bare iron oxide (Fe_3_O_4_) NPs, a room temperature modified Massart method was applied, which was derived from the work carried out by Popescu et al. [22]: an aqueous solution of ferrous sulfate and ferric chloride, [FeSO_4_•7H_2_O]:[FeCl_3_] = 1:1.6 (Sigma Aldrich Chemie GmbH, Darmstadt, Germany) was added drop wise into a basic medium. The resulted NPs were washed several times and suspended in ultrapure water at a final concentration of 4 mg/mL. In order to obtain the functionalized NPs (Fe_3_O_4_@PEG 20 K and Fe_3_O_4_@PEG 35 K, respectively), an in situ method was applied by introducing polyethylene glycol with molecular weight of 20,000 Da and, respectively, 35,000 Da (Sigma Aldrich Chemie GmbH, Darmstadt, Germany) into the precipitation medium (mixing into the basic solution) at a concentration of 0.5% w/vol.

TEM, HR-TEM, and SAED analyses were performed using a Tecnai G2 F30 S-TWIN HR-TEM (FEI Company, Hillsboro, OR, USA) equipped with SAED. The samples were prepared for analysis by successive dilutions in ultrapure water followed by sonication, to obtain suitable concentrations for visualization. The final suspension was placed onto a holey carbon–copper grid and dried. The investigations were carried out in transmission mode, at 300 kV, with a point resolution of 2 Å and line resolution of 1 Å.

The size, circularity, and roundness of the nanoparticles were determined using the ImageJ (National Institute of Health, Bethesda, MD, USA) software. For this, several images containing transmission electron microscopy micrographs were loaded in the program. Based on the scale of the images, a measuring scale was selected. Using the drawing tools (manual line or freehand selections), the diameter of each nanoparticle was measured (in two to three points for each nanoparticle) and, respectively, the perimeter of each nanoparticle was surrounded. A total of about 500 nanoparticles were measured for each type of sample. The circularity and roundness of the nanoparticles were automatically determined by the software, the value 1.0 indicating a perfect circle.

The Zeta Potential of the NPs was measured using the Delsa Nano C (Beckman Coulter, Brea, CA, USA) and the recording was carried out with DelsaNano 3.73 software (Beckman Coulter, Brea, CA, USA) for stock solutions.

The in vivo biocompatibility and biodistribution of the Fe_3_O_4_, Fe_3_O_4_@PEG 20 K, and Fe_3_O_4_@PEG 35 K, respectively, were performed on three-month-old BALB/c male mice. The experimental protocol was carried out in concordance with the European Council Directive No. 86/609, the European Convention on Protection of Vertebrate Animals (2005) and National Law No. 43/2014 regarding the protection of animals used for scientific purposes. The study was approved by the Ethics Committee of the University of Medicine and Pharmacy of Craiova, Romania (Approval No. 118/27.05.2015). The mice were aseptic and intravenously injected in the left jugular vein using 100 μL of 1 mg/mL NPs sterile suspension in saline. Control mice were injected with 100 μL of saline solution. The administration was carried out under general anesthesia. Control mice were administered 100 μL saline under the same conditions. During the examination period, the mice were housed in the Animal Care Unit of the University of Medicine and Pharmacy of Craiova, maintained in standard conditions (22 ± 2 °C, 55 ± 10% humidity and a 12 h light–dark cycle, water and food ad libitum). At 2 and 10 days after the administration of nanoparticle treatment, the animals were euthanized, and the main organs were harvested and prepared for histopathological examination (brain, liver, heart, pancreas, lungs, kidney, and spleen). Microscopy samples were prepared from 4 μm thick slices stained using Hematoxylin–Eosin and analyzed with a Nikon Eclipse 55i microscope equipped with a DS-Fil CCD camera (Nikon Instruments, Apidrag, Romania).

## 3. Results and Discussion

This study presents the synthesis, characterization, and in vivo biodistribution of bare IONPs and PEG-conjugated, respectively, obtained using a novel modified Massart chemical coprecipitation method [22]. The addition of the polymer in the system was carried out through a “one pot” high yield synthesis approach. The influence of the polymer molecular weights on the morphology, structure, and in vivo biocompatibility was evaluated.

**Figure 1:**
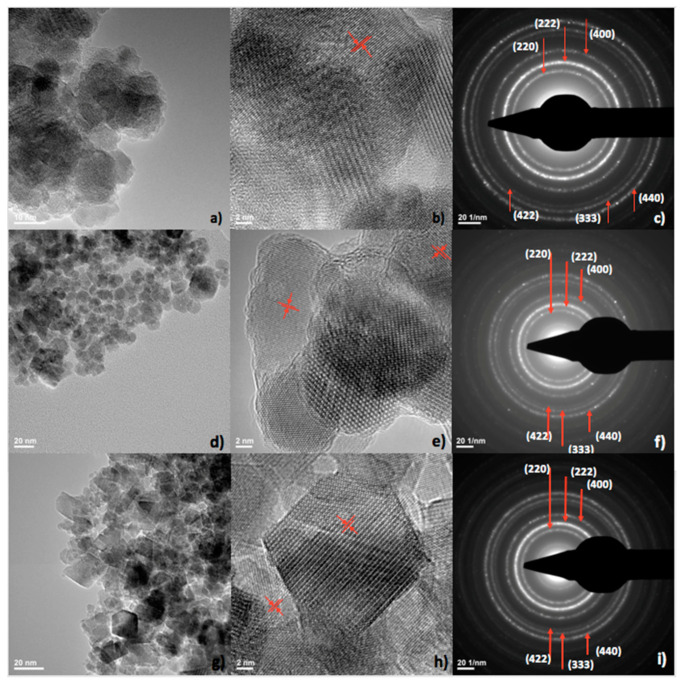
Transmission electron microscopy (TEM) images (**a**,**d**,**g**), high resolution TEM (HR-TEM) (**b**,**e**,**h**), respectively, selected area electron diffraction (SAED) spectrums (**c**,**f**,**i**) for Fe_3_O_4_ (**a**–**c**), Fe_3_O_4_@PEG 20 K (**d**–**f**) and Fe_3_O_4_@PEG 35 K (**g**–**i**). Red arrows in (**b**,**e**,**h**) indicate (220) crystalline plane.

The TEM technique was used to evaluate the morphology of the functionalized and non-functionalized IONPs (Figure 1a,d,g). Thus, bare IONPs highlighted a round shape, having physical dimensions between 11.6 ± 3.6 nm (circularity = 0.869, roundness = 0.844). Similarly, round-shaped PEG 20 K functionalized nanoparticles have diameters of 12.9 ± 4.02 nm (circularity = 0.893, roundness = 0.837). The morphology of the NPs was severely affected upon conjugation with PEG 35 K, resulting in NPs with larger diameters (17.5 ± 10.5 nm) and rhombic shape (circularity = 0.789, roundness = 0.747). However, some very small, round-shaped NPs could be observed, probably due to an incomplete or absent functionalization.

Moreover, HR-TEM gave information on the level of crystallinity and structural characteristics of the samples (Figure 1b,e,h). The (220) crystalline plane of 0.29 nm, characteristic of the mineralogical phase magnetite was evidenced for all samples, Fe_3_O_4_, Fe_3_O_4_@PEG 20 K and Fe_3_O_4_@PEG 35 K, respectively (Figure 1b,e,h—red arrows) [22,23]. SAED analysis confirmed the high crystallinity of the samples and emphasized diffraction rings characteristic for spinel structured face-centered magnetite phase: (220), (222), (400), (440), (333), (422) (Figure 1c,f,i) [22,23].

Conjugation of Fe_3_O_4_ and PEG polymers was evidenced through Zeta Potential measurements of the stock solutions by an increase in stability. In the case of bare nanoparticles, a value of −0.01 mV showed instability in water-based mediums. However, the value decreased with the introduction of the polymer in the system (−17.74 mV in the case of Fe_3_O_4_@PEG 35 K and, respectively, −19.38 mV for Fe_3_O_4_@PEG 20 K) and thus the PEG- conjugated NPs stability increased [31]. The binding mechanism, in this case, is mainly carried out through non-covalent binding, based on hydrogen bonds between the –OH functional groups of the iron oxide and polymer and favored by the basic pH reaction conditions [32].

Fe_3_O_4_, Fe_3_O_4_@PEG 20 K, and Fe_3_O_4_@PEG 35 K NPs were intravenously injected into mice model to evaluate their distribution at 2 and 10 days after administration by comparison. The biodistribution study in healthy subjects is a measure of the toxicity of NPs and can provide information on their biocompatibility and clearance mechanisms. The time points selected for this investigation are longer than the half-life of PEG and/or iron oxide nanoparticles [33,34,35,36] but shorter than long term investigations on this topic, covering a timeframe that is not particularly reviewed by the scientific literature. Moreover, the administered dose (4 mg NPs/kg) is the equivalent used in clinical investigations of diagnosis for PEG- iron oxide systems [36,37].

**Figure 2:**
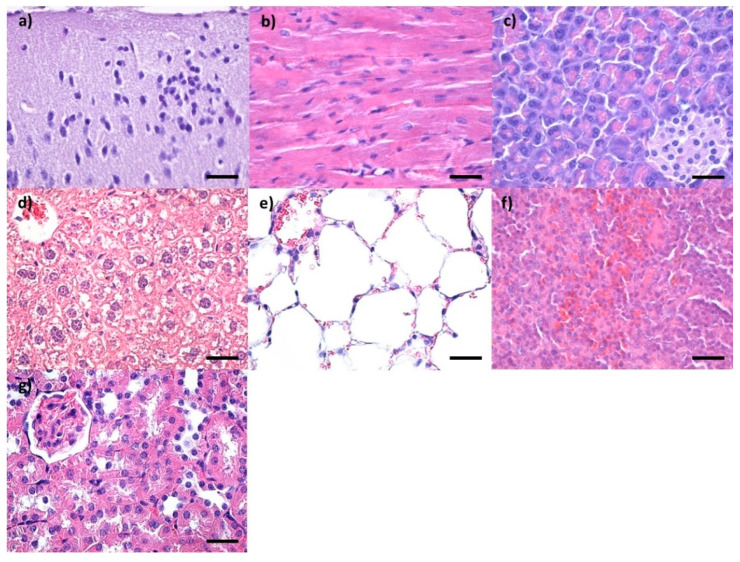
Cross-section through (**a**) brain, (**b**) myocardium, (**c**) pancreas, (**d**) liver, (**e**) lungs, (**f**) spleen and (**g**) kidney from control mice injected with saline solution and harvested at 2 days after intravenous administration; normal morphology; Hematoxylin–Eosin staining; 400× magnification, scale bar 50 μm.

**Figure 3:**
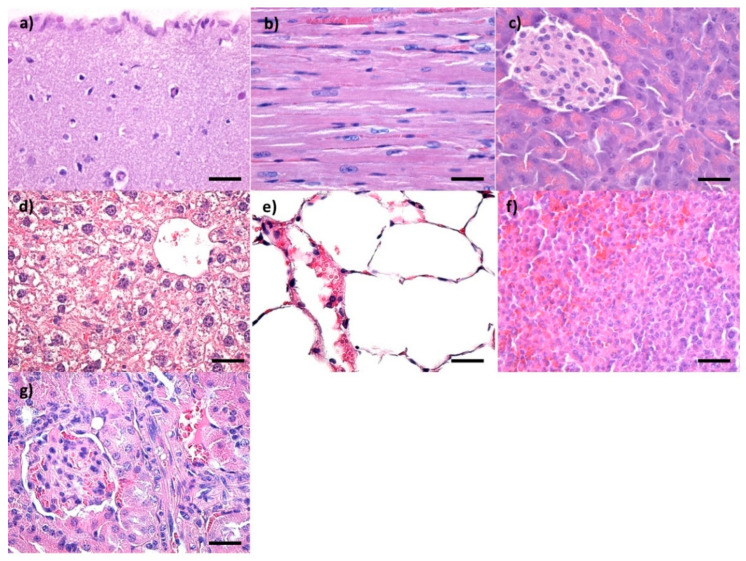
Cross-section through (**a**) brain, (**b**) myocardium, (**c**) pancreas, (**d**) liver, (**e**) lungs, (**f**) spleen, and (**g**) kidney from control mice injected with saline solution and harvested at 10 days after intravenous administration; normal morphology; Hematoxylin–Eosin staining; 400× magnification, scale bar 50 μm.

Control mice were administered saline solution and subjected to the same protocol. No alterations in the histological appearance of the main organs were observed (Figure 2 and Figure 3).

**Figure 4:**
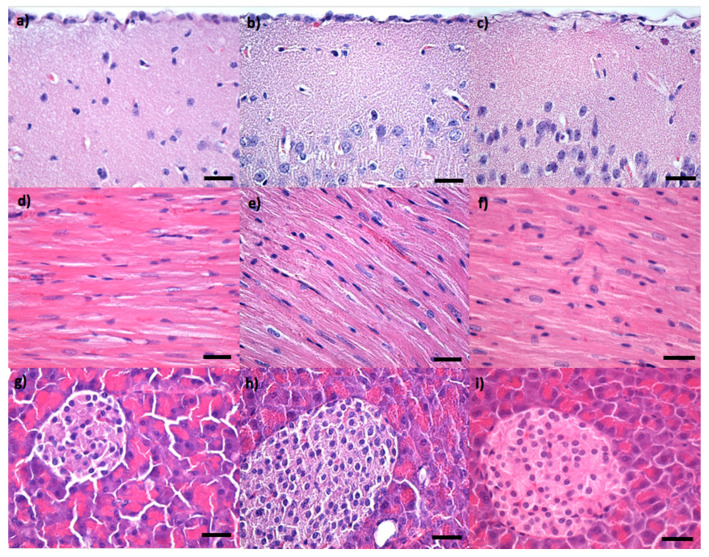
Cross-section through (**a**–**c**) brain, (**d**–**f**) myocardium, (**g**–**i**) pancreas from mice injected with Fe_3_O_4_ (**a**,**d**,**g**), Fe_3_O_4_@PEG 20 K (**b**,**e**,**h**) and Fe_3_O_4_@PEG 35 K NPs (**c**,**f**,**i**), respectively, and harvested at two days after intravenous administration; normal morphology; Hematoxylin–Eosin staining; 400× magnification, scale bar 50 μm.

**Figure 5:**
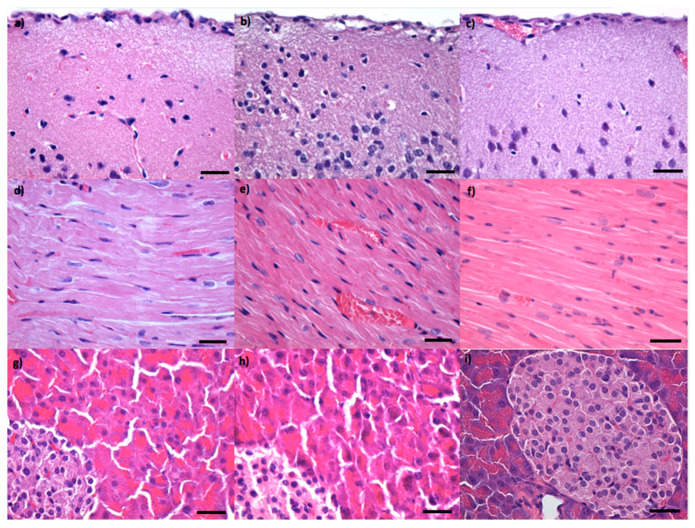
Cross-section through (**a**–**c**) brain, (**d**–**f**) myocardium, and (**g**–**i**) pancreas from mice injected with Fe_3_O_4_ (**a**,**d**,**g**), Fe_3_O_4_@PEG 20 K (**b**,**e**,**h**) and Fe_3_O_4_@PEG 35 K NPs (**c**,**f**,**i**), respectively, and harvested at 10 days after intravenous administration; normal morphology; Hematoxylin–Eosin staining; 400× magnification, scale bar 50 μm.

Neither at 2 nor at 10 days after treatment could the presence of any of the NPs involved in the study be detected in the brain (Figure 4a–c and Figure 5a–c), and no histopathological alterations of the nervous tissue could be observed. This suggested the fact that none of the Fe_3_O_4_-based IONPs can cross the blood-brain barrier. Moreover, Fe_3_O_4_, Fe_3_O_4_@PEG 20 K, and Fe_3_O_4_@PEG 35 K NPs were not evidenced in the myocardium (Figure 4d–f and Figure 5d–f) or pancreas (Figure 4g–i and Figure 5g–i) at 2 and 10 days after intravenous injection. Although the absence of the nanoparticle aggregates is emphasized at the micrometric and submicrometric levels, this does not exclude the fact that nanosized aggregates or single nanoparticles can still be present at the subcellular level. The purpose of this study was to give information on the biodistribution of IONPs at tissue level.

**Figure 6:**
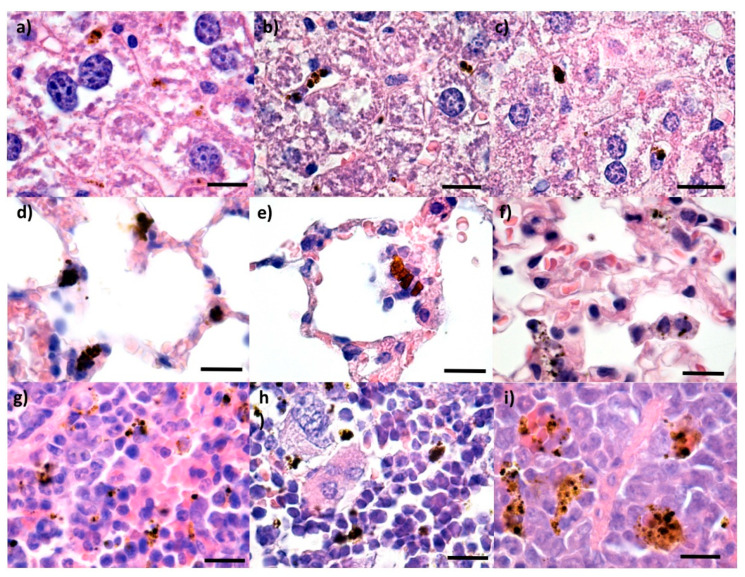
Cross-section through the (**a**–**c**) liver, (**d**–**f**) lungs, and (**g**–**i**) spleen from mice injected with Fe_3_O_4_ (**a**,**d**,**g**), Fe_3_O_4_@PEG 20 K (**b**,**e**,**h**) and Fe_3_O_4_@PEG 35 K NPs (**c**,**f**,**i**), respectively, and harvested at 2 days after intravenous administration; normal morphology; Hematoxylin–Eosin staining; 1000× magnification, scale bar 25 μm.

**Figure 7:**
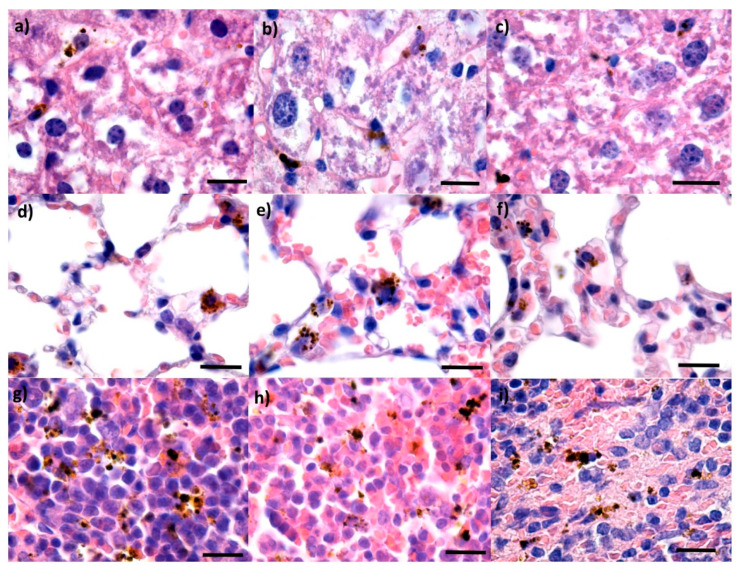
Cross-section through the (**a**–**c**) liver, (**d**–**f**) lungs, and (**g**–**i**) spleen from mice injected with Fe_3_O_4_ (**a**,**d**,**g**), Fe_3_O_4_@PEG 20 K (**b**,**e**,**h**) and Fe_3_O_4_@PEG 35 K NPs (**c**,**f**,**i**), respectively, and harvested at 10 days after intravenous administration; normal morphology; Hematoxylin–Eosin staining; 1000× magnification, scale bar 25 μm.

At two days after treatment, for all three IONP groups, the presence of NPs was evidenced as dark brown aggregates in low concentration in both blood vessels as well as in the Kupffer cells situated at the periphery of the hepatic sinusoidal capillaries in liver (Figure 6a–c). The density of NPs in the Kupffer cells was variable from one cell to another and directly proportional to the dimension of the hepatic sinusoidal capillary in the hepatic parenchyma. At 10 days from intravenous administration (Figure 7a–c), the NPs could still be observed in the hepatic histological structures. No obvious additional changes in the density of NPs or histopathological alterations could be noticed.

At the lungs level, for all three samples at two days from administration (Figure 6d–f), NPs were mainly evidenced in perivascular macrophages and the macrophages at the level of the interalveolar septum. The IONPs density was different depending on the type of cells: the highest IONPs density was observed for perivascular macrophages, while the lowest density was found for the macrophages in the intra-alveolar septum. The NPs were also evidenced in the intravascular cells of the monocyte-macrophage system. This fact and the presence of the NPs in the vascular lumen could be explained by the fact that the monocytes’ precursor cells of the macrophages in the red bone marrow, have internalized IONP structures. Moreover, in the vascular lumen, isolated extracellular NP aggregates were evidenced, possibly in blood platelets (Figure 6e). Similar observations were made for the samples harvested 10 days after treatment (Figure 7d–f).

In the spleen, all three types of NPs could be observed at two days as dark brown spherical aggregates located in the red pulp (Figure 6g–i). The IONPs were absent from the white pulp. However, white pulp hypertrophy was evidenced at two days, since the NPs have stimulated the formation of macrophages in the multilobular nuclei. In the red pulp, the IONPs were evidenced at the level of the macrophage system cells, but also in the Billroth cords and the sinusoidal capillaries. The NPs were present as granular structures with variable dimensions up to 3 μm. The density was variable from one cell to another, with some of them displaying a higher quantity of internalized NPs.

At 10 days after the treatment, Fe_3_O_4_, Fe_3_O_4_@PEG 20 K, and Fe_3_O_4_@PEG 35 K NPs were as well evidenced in the red pulp, in the macrophage system cells, Billroth cords and sinusoid capillaries, but not the white pulp (Figure 7g–i). A higher density of variable- sized black aggregates was noticed in the samples harvested at this time, compared to the ones analyzed at 2 days after administration.

**Figure 8:**
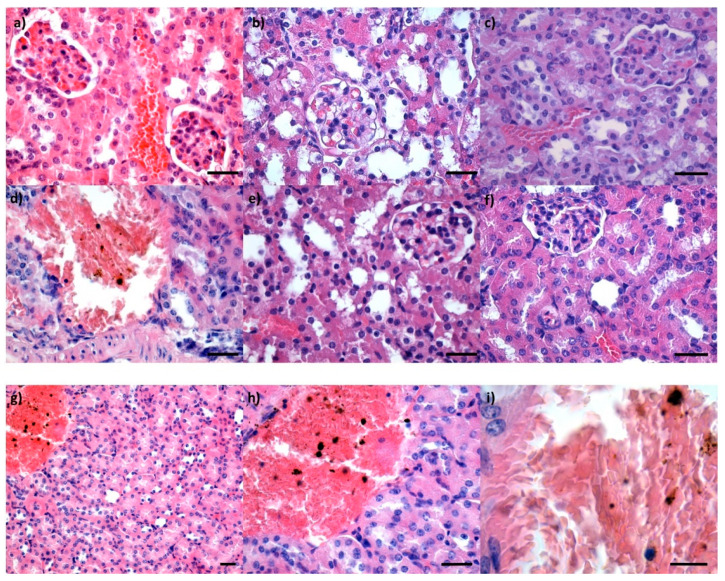
Cross-section through a kidney from mice injected with Fe_3_O_4_ (**a**,**d**), Fe_3_O_4_@PEG 20 K (**b**,**e**) and Fe_3_O_4_@PEG 35 K NPs (**c**,**f**), respectively, and harvested at 2 days (**a**–**c**) and 10 days (**d**–**f**), respectively, after intravenous administration; Hematoxylin–Eosin staining; 400× magnification, scale bar 50 μm; figures (**g**–**i**) represent details of cross-section through kidney from mice injected with Fe_3_O_4_ NPs and harvested at 10 days after intravenous administration; Hematoxylin–Eosin staining; different magnifications: (**a**) 200×, scale bar 50 μm; (**b**) 400×, scale bar 50 μm; (**c**) 1000×, scale bar 25 μm.

The presence of none of Fe_3_O_4_, Fe_3_O_4_@PEG 20 K and Fe_3_O_4_@PEG 35 K NPs, respectively, was evidenced after 2 days from administration in renal parenchyma: neither in glomeruli, renal tubes, or renal stroma (Figure 8a–c). However, at 10 days from treatment, a low concentration of NP aggregates was observed in the kidney blood vessels, only for Fe_3_O_4_ NP samples, showing that renal clearance is a possible way to eliminate this type of NPs (Figure 8d,g–i). The in situ PEG conjugation of the NPs delays or prevents their elimination through the renal system.

The main clearance pathway in the case of IONPs is the mononuclear phagocyte system (reticuloendothelial system), with cells located predominantly in the liver, spleen and fewer in the lungs [38]. All IONPs involved in this study, namely Fe_3_O_4_, Fe_3_O_4_@PEG 20 K, and Fe_3_O_4_@PEG 35 K NPs, respectively, have been detected in these organs since the second day after intravenous injection. The NPs were identified inside cells such as circulating monocytes (which usually extravagate from the blood stream inside different areas) and macrophages (which are naturally stored in these organs). Moreover, monocytes, which are precursors of macrophages, can be produced in the bone marrow and extravagate into the tissues when needed.

As evidenced by our investigations, IONPs were located in the liver inside the Kupffer cells and in alveolar macrophages in the lungs, which was in concordance with observations made by other research groups [39,40,41]. The presence of IONPs in the lungs was explained by the excess of NPs in the bloodstream. The presence of IONPs in Kupffer cells and reticular macrophages in the liver is a sign of elimination through the opsonization phenomenon [42,43].

The elimination of IONPs through the macrophages in the spleen is a secondary filtering barrier for this type of NPs [44,45]. Our results emphasized the localization of IONPs in the red pulp, due to the extraversion of macrophages carrying NPs in the porous capillaries at the extremity of these regions.

The macrophages in the pulmonary, hepatic, splenic level specialize in capturing of specific antigens in the local environment. Thus, a difference in the NP capturing by macrophages at this level is given by a difference in the reaction intensity.

The PEGylation of IONPs has not only been proved to increase the half-time of NPs in the body [46] but also to determine their elimination through a different pathway. Cole et al. [47] have shown that PEG cross-linked IONPs have been predominantly cleared through the macrophages in the spleen than through specialized cells in the liver and were correlated with longer blood life and higher hydrodynamic dimension. Renal clearance in case of PEGylated NPs is less possible since the higher dimension prevented the passage through the kidney fenestrations [21].

However, depending on the application, the macrophage uptake can be either beneficial or not. For example, it can be good in certain cases of diagnosis and visualization, such as disorders associated with inflammation, but in applications of cancer visualization or vascular angiography it is not desired [48].

## 4. Conclusions

In this study, we have successfully prepared iron oxide (Fe_3_O_4_) NPs using a modified chemical co-precipitation method and have functionalized them in situ using PEG with different molecular weights (20 K and 35 K, respectively).

Morphological investigations using TEM revealed that the dimension of the NPs increases with the polymer shell’s molecular weight and also their diameter distribution is larger. Functionalization of Fe_3_O_4_ with PEG 35 K induces a rhombic morphology to the resulting NPs. However, very small round IONPs are also present.

Structural investigations were carried out using HR-TEM and SAED, revealing the high level of crystallinity of the resulting spinel structured cubic face-centered magnetite NPs.

The in vivo biological assessment proved the biocompatibility of these NPs, as no major histopathological alterations were observed in the examined tissues. The investigations showed that the IONPs are cleared through the immune cells in the liver, spleen, and lungs. The presence of bare IONPs was evidenced in the blood vessels in the kidneys.
